# Validation of the European Cross-Cultural Neuropsychological Test Battery (CNTB) for the assessment of mild cognitive impairment due to Alzheimer's disease and Parkinson's disease

**DOI:** 10.3389/fnagi.2023.1134111

**Published:** 2023-05-05

**Authors:** Alfonso Delgado-Álvarez, Thomas Rune Nielsen, Cristina Delgado-Alonso, María Valles-Salgado, Juan I. López-Carbonero, Rocío García-Ramos, María José Gil-Moreno, María Díez-Cirarda, Jorge Matías-Guiu, Jordi A. Matias-Guiu

**Affiliations:** ^1^Department of Neurology, Hospital Clinico San Carlos, San Carlos Institute for Health Research (IdISSC), Universidad Complutense, Madrid, Spain; ^2^Faculty of Psychology, Universidad Autónoma de Madrid, Madrid, Spain; ^3^Department of Neurology, Danish Dementia Research Centre, University of Copenhagen-Rigshospitalet, Copenhagen, Denmark

**Keywords:** Alzheimer's disease, cognitive assessment, cross-cultural neuropsychology, mild cognitive impairment, Parkinson's disease

## Abstract

**Background:**

The Cross-Cultural Neuropsychological Test Battery (CNTB) is a novel test battery specifically designed to reduce the impact of multiculturality in cognitive assessment.

**Objective:**

We aimed to validate the CNTB in Spaniards in patients with Alzheimer's disease (AD), including patients at mild cognitive impairment (MCI) and mild dementia stages, and Parkinson's disease with MCI (PD-MCI).

**Methods:**

Thirty patients with AD-MCI, 30 with AD-dementia (AD-D), and 30 with PD-MCI were recruited. Each clinical group was compared against a healthy control group (HC) with no differences in sex, age, or years of education. Intergroup comparisons, ROC analysis, and cut-off scores were calculated.

**Results:**

AD-MCI scored lower than HC in those subtests associated with episodic memory and verbal fluency. AD-D also showed lower scores in executive functions and visuospatial tests. Effect sizes for all the subtests were large. PD-MCI showed lower performance than HC in memory and executive functions, particularly on error scores, with large effect sizes. Comparing AD-MCI and PD-MCI, AD-MCI had lower memory scores, while PD-MCI showed the worst performance in executive functions. CNTB showed appropriate convergent validity with standardized neuropsychological tests measuring the same cognitive domains. We obtained similar cut-off scores to previous studies performed in other populations.

**Conclusions:**

The CNTB showed appropriate diagnostic properties in AD and PD, including those stages with mild cognitive impairment. This supports the utility of the CNTB for the early detection of cognitive impairment in AD and PD.

## 1. Introduction

The study of cross-cultural neuropsychological assessment has focused on the relationship between cognitive assessments and possible cultural biases that may have an impact on test scores and their interpretations. The identification of cultural variables, such as patterns of abilities, familiarity, acculturation, and language (Rosselli et al., 2022) allows the development and application of cross-cultural instruments to improve cognitive assessment quality in multicultural settings. In this regard, the European Consortium on Cross-Cultural Neuropsychology (ECCroN) has recently highlighted the need for new cross-cultural tools, considering the different migratory movements that are taking place nowadays (Franzen et al., 2021, 2022).

While some progress has been made in the screening test field, more studies are necessary in the context of comprehensive neuropsychological batteries. Some of the most important cross-cultural screening tests are the Rowland Universal Dementia Assessment Scale (RUDAS; Storey et al., 2004), the Cross-Cultural Dementia screening test (CCD; Goudsmit et al., 2017), the Multicultural Cognitive Examination (MCE; Nielsen et al., 2019a), and the Brief Assessment of Impaired Cognition (BASIC; Jørgensen et al., 2022). Recently, a novel neuropsychological battery has been proposed as a comprehensive neuropsychological instrument: the European Cross-cultural Neuropsychological Test Battery (CNTB; Nielsen et al., 2019b).

CNTB allows the assessment of global cognition function, memory, language, executive functions, and visuospatial functions based on a cross-cultural approach that reduces the impact of cultural variables (Nielsen et al., 2018; Nielsen, 2019; Al-Jawahiri and Nielsen, 2021). The battery has been validated in a Western European cross-sectional multi-center study, including 66 patients with dementia (62% minority, 38% majority background) and 118 healthy control participants (44% minority and 56% majority background; Nielsen et al., 2019b). The clinical sample included participants with cognitive impairment due to Alzheimer's disease (AD; *n* = 35), cerebrovascular disease (*n* = 4), mixed AD and cerebrovascular disease (*n* = 18), Lewy bodies/Parkinson's disease (PD; *n* = 3), frontotemporal lobar degeneration (*n* = 3), normal pressure hydrocephalus (*n* = 2), and cognitive impairment related to exposure to organic solvents, stroke, and anoxia (*n* = 1; Nielsen et al., 2019b). The study described promising cross-cultural diagnostic properties for assessment of dementia in minority and majority populations.

In the same line, the Brazilian version of the CNTB was validated in a study including 70 participants with AD at mild and moderate stages of the disease [Clinical Dementia Rating (CDR) of 1.0 or 2.0] and 56 healthy control participants, showing good psychometric properties for the correct classification of participants, particularly considering the scores on the Recall of Pictures Test and Enhanced Cued Recall test (Araujo et al., 2020).

The performance of majority and minority cognitively intact participants has been previously reported (Nielsen et al., 2018); however, with a low representation of majority population from the South of Europe.

AD is the most common cause of dementia (Mayeux and Stern, 2012) and episodic memory deficits are the main cognitive symptoms at early stages, followed by changes in functioning and behavior (Dubois et al., 2016). Among all dementia types, AD has been the main target of the CNTB validation studies, but mainly at the dementia stages. Furthermore, only three patients with PD were included in the original validation of CNTB, also at the dementia stage. PD is the second most frequent neurodegenerative disorder and is commonly associated with cognitive impairment (Poewe et al., 2017). Motor disorders are the hallmark of PD, but the relevance of cognitive deficits are increasingly noticed, including executive functions, attention, visuospatial skills, and memory deficits (Muslimovic et al., 2005).

To our knowledge, there are no validation studies at the early prodromal stages of AD, in which cross-cultural tools are urgently needed (Matias-Guiu and Delgado-Álvarez, 2022), or PD with mild cognitive impairment. Furthermore, cognitive performance on CNTB has not previously been described in Southern European countries. In particular, Spaniards may be of interest, considering Spanish is one of the most spoken languages in the world. Thus, we aimed to validate the CNTB in Spaniards and, as a novelty, we focused on people with AD, including patients at early stages of the disease, and PD with mild cognitive impairment. In addition, we compared scores on CNTB between AD and PD with mild cognitive impairment.

We hypothesized that CNTB may depict different cognitive profiles according to each disease and may allow a correct classification between healthy controls and people with AD and PD at mild cognitive impairment stages.

## 2. Materials and methods

### 2.1. Participants

A total of 150 participants were enrolled in this study. All participants were Caucasians, Spaniards, and monolinguals (Spanish as their mother tongue). Main clinical and demographic characteristics are described in Table 1. The recruitment process was carried out at the Department of Neurology of the Hospital Clínico San Carlos between 2019 and 2021. The AD group was composed of 30 participants with a CDR score of 1.0 (AD-Dementia, AD-D) and 30 participants with a CDR score of 0.5 (AD-Mild Cognitive Impairment, AD-MCI). The PD group involved 30 participants with mild cognitive impairment (PD-MCI). In addition, 60 healthy controls (HC) were recruited (30 for intergroup comparisons with AD, and 30 for comparisons with PD-MCI). There were no statistically significant differences between each HC group and the corresponding clinical group in sex, age, or years of education (Supplementary material 1).

**Table 1:** Main demographic and clinical characteristics of all groups.

	**Alzheimer's disease**			**Parkinson's disease**	
	**AD-MCI**	**AD-D**	**HC**	**PD-MCI**	**HC**
N	30	30	30	30	30
Sex, female %	73.3%	73.3%	60%	23.3%	46.7%
Age, years	76.20 (5.85)	76.63 (5.56)	77.37 (5.22)	70.33 (8.68)	67.67 (10.57)
Years of education	7.10 (2.75)	7.03 (3.38)	6.83 (3.86)	11.20 (4.76)	8.90 (4.21)
GDS	0.33 (0.76)	0.73 (0.94)	0.20 (0.66)	0.72 (0.92)	0.13 (0.57)
FAQ	4.33 (3.54)	10.96 (6.34)	0	–	0
IDDD	37.10 (4.24)	42.96 (4.71)	–	–	–
SCOPA-COG	–	–	–	20.43 (6.21)	–
Hoehn & Yahr	–	–	–	2.05 (0.34)	–

GDS, Geriatric depression scale; FAQ, Functional activities questionnaire; IDDD, Interview for deterioration in daily living activities in dementia; Hoehn and Yahr, Hoehn and Yahr scale; AD-MCI, Alzheimer's disease with mild cognitive impairment group; AD-D, Alzheimer's disease with dementia group; HC, Healthy control group; PD-MCI, Parkinson's disease with mild cognitive impairment group.

The inclusion criteria for AD were as follows: (1) complaints of memory loss with CDR of 1.0 and at least 0.5 in memory box for AD-D group and CDR of 0.5 and 0.5 in memory box for AD-MCI group, (2) presence of biomarkers supporting the diagnosis of AD (FDG-PET: temporoparietal hypometabolism and/or cerebrospinal fluid: altered A-beta 1–42, tau and phosphotau levels), (3) confirmation of clinical progression during the follow-up (Albert et al., 2011). The inclusion criteria for PD-MCI were as follows: (1) diagnosis of PD following the criteria of the Movement Disorder Society (MDS), (2) evidence Level II (comprehensive cognitive assessment) of mild cognitive impairment (MCI) according to criteria (Litvan et al., 2012). Exclusion criteria for the clinical sample were as follows: (1) prior history of any medical, neurological, or psychiatric comorbidity with a negative impact on the test performance, (2) physical limitations (e.g., hearing or visual problems) that could bias the neuropsychological tests.

For the HC group, the inclusion criteria were: CDR = 0 and absence of functional impairment assessed by Functional Activities Questionnaire (FAQ) scores = 0 (Olazarán et al., 2005). The exclusion criteria were: (1) prior or current history of neurological or psychiatric disease, (2) any medical disorder associated with cognitive impairment, (3) any physical difficulties (e.g., hearing or visual problems) that could bias the neuropsychological tests.

### 2.2. Neuropsychological assessment

The clinical groups completed the comprehensive neuropsychological battery Neuronorma (NN) with normative data in our setting (Peña-Casanova et al., 2009) and the Geriatric Depression Scale (GDS; Yesavage et al., 1982). In addition, the AD groups were assessed using the CDR scale for a correct classification between AD-D and AD-MCI and the Interview of Deterioration in Daily living activities in Dementia (IDDD; Böhm et al., 1998), while the PD-MCI group was evaluated using the Scales for Outcomes in Parkinson's Disease-Cognition (SCOPA-COG; Marinus et al., 2003) and the Hoehn and Yahr (1967). The HC groups completed the FAQ, CDR, and GDS to ensure the inclusion criteria.

All participants completed the CNTB (Nielsen et al., 2019b), which includes the Recall of Pictures Test (RPT), Enhanced Cued Recall test (ECR), semantic fluency tasks (“animals” and “supermarket”), a naming task, Color Trails Test (CTT), Five Digit Test part 1, 2, and 3 (FDT), serials threes, copying of simple figures (SF copy), copying of a semi-complex figure (SCF copy), delayed recall (3 min) of the SCF, Clock Drawing Test (CDT), and Clock Reading Test (CRT). All tests are described in Supplementary material 2. No cultural or language adaptation procedure was required to our context. Scores on the CNTB were not considered for the diagnosis. The results of the screening test RUDAS included in the CNTB have been previously reported (Delgado-Álvarez et al., 2022c).

### 2.3. Procedure

The study had the approval of our hospital's Ethics Committee (code 19/126-E), and all participants gave written informed consent. All cognitive assessments were performed by trained neuropsychologists in two independent sessions with a total duration of ~3 h.

In the AD groups, first CDR and FAQ were completed for the correct classification between AD-D and AD-MCI. In PD, MCI was defined when at least two scaled scores ≤ 5 (adjusted for age and years of education) in one or more cognitive domain according to the NN battery. All PD-MCI participants were evaluated in their optimal motor state, following the recommendations of the MDS (Litvan et al., 2012).

### 2.4. Sample size

We estimated that a sample size of at least 56 subjects for comparing two groups (*t*-test; PD-MCI vs. HC) and 84 for comparing three groups (ANOVA; AD-D, AD-MCI, and HC) was needed to obtain more than 90% power for detecting large effect sizes (d = 0.8, f = 0.4) with an alpha error < 0.05 (two-tails). These effect sizes were selected according to previous studies using CNTB (where Cohen's d > 2 was found in many subtests in the comparison between dementia and controls) (Nielsen et al., 2019b) and considering the values needed for clinical application (Bezeau and Graves, 2001).

### 2.5. Statistical analysis

Statistical analysis was performed using SPSS Statistics 28.0. Alpha was set at 0.05 and Bonferroni correction was applied for multiple comparisons. For the study of normality, Shapiro–Wilk test was calculated.

For intergroup comparisons, Pearson's chi-squared test was calculated for categorical variables. Kruskal–Wallis and *post-hoc* tests were calculated for quantitative variables and in those cases with more than two groups, while Mann–Whitney *U* was calculated for comparisons between two groups. As effect size measures, eta squared (small = 0.01, medium = 0.06, and large = 0.14), Cohen's d (small = 0.20, medium = 0.50, and large = 0.80), and *r* (small = 0.30, medium = 0.50, and large = 0.70) were considered.

The relationship between quantitative variables was examined using Spearman's rho correlation and was categorized as very low (0–0.29), low (0.30–0.49), moderate (0.50–0.69), high (0.70–0.89), or very high (>0.89).

For those variables with significant differences between groups (clinical vs. HC), ROC analysis was conducted and reported when the area under the curve (AUC) was >0.70. Cut-off scores were established based on Youden's index (>0.40). Sensitivity, specificity, positive predictive value (PPV), and negative predictive value (NPV) were reported for our sample. In addition, PPV and NPV at different base rate were calculated, considering the prevalence of primary care or general hospital settings (25%) and high prevalence settings, such as memory clinics (50%; Larner and Mitchell, 2014; Supplementary materials 3–6).

To compare the AD-MCI and PD-MCI groups, ANCOVA models were used to control for sex, age, and years of education.

## 3. Results

### 3.1. Alzheimer's disease: AD-MCI, AD-D, and HC

#### 3.1.1. Intergroup comparisons

Patients with AD-MCI showed lower scores on the memory measures of RPT, ECR, and recall of SCF, semantic fluency, FDT and serials threes compared with HC. Effect sizes were large for all the tests. Patients with AD-D, in comparison with controls, showed lower scores on the same tests as AD-MCI as well as on CTT 1, FDT3 (time), copying of SCF, CDT, and CRT, also with large effect sizes (Table 2).

**Table 2:** Scores and differences between AD and HC groups on CNTB tests.

**CNTB measure**	**AD-MCI *n* = 30**	**AD-D *n* = 30**	**HC *n* = 30**	F^**^/*H* (*p*)	**η^2^**
**Memory**					
RPT 1st recall (/10)	2.43 (1.69)	1.48 (1.02)	3.79 (1.68)	27.09 (< 0.001)^a, b^^*^	0.29
RPT total recall (/30)	13.67 (4.02)	9.21 (3.72)	18.07 (4.51)	30.79 (< 0.001)^a, b, c^^*^	0.42
RPT delayed recall (/10)	2,60 (2.46)	0.76 (1.57)	5.54 (2.20)	42.24 (< 0.001)^a, b, c^^*^	0.46
RPT recognition (/10)	8.63 (1.54)	6.86 (1.64)	9.46 (0.99)	31.54 (< 0.001)^b, c^^*^	0.34
ECR free recall (/16)	2.70 (1.86)	1.93 (1.44)	6.39 (2.33)	44.55 (< 0.001)^a, b^^*^	0.51
ECR total recall (/16)	9.60 (3.83)	5.86 (3.62)	14.36 (1.44)	50.12 (< 0.001)^a, b, c^^*^	0.55
Recall of SCF (/22)	7.93 (5.90)	1.76 (2.74)	15.07 (4.22)	47.10 (< 0.001)^a, b, c^^*^	0.52
**Language**					
Naming (/10)	9.80 (0.48)	9.66 (0.67)	9.89 (0.31)	2.82 (0.244)	< 0.01
Verbal fluency “animals”	11.80 (4.20)	9.10 (3.40)	15.64 (4.53)	18.54 (< 0.001)^a, b, c^^*^	0.31
Verbal fluency “supermarket”	13.97 (4.02)	12.20 (4.98)	19.43 (5.47)	17.34 (< 0.001)^a, b^^*^	0.29
**Attention and EF**					
CTT 1 (seconds)^+^	81.81 (46.94)	144.7 (69.18)	78.86 (33.21)	17.98 (< 0.001)^b, c^^*^	0.18
CTT 2 (seconds)^+^	182.4 (66.39)	245.56 (79.78)	184.61 (67.64)	5.06 (0.080)	0.03
CTT 2 (errors)	0.50 (1.10)	2.00 (1.50)	0.50 (0.84)	10.61 (0.005)	0.09
FDT 1 (seconds)^++^	33.33 (10.05)	38.0 (14.11)	30.82 (7.78)	4.36 (0.113)	0.02
FDT 2 (seconds)^++^	34.57 (11.87)	37.5 (14.52)	31.43 (7.81)	2.57 (0.276)	0.006
FDT 3 (seconds)^++^	70.20 (36.56)	84.38 (45.53)	51.25 (11.86)	16.12 (< 0.001)^**b**^	0.16
FDT 3 (errors)	4.37 (3.77)	6.23 (4.33)	1.54 (2.28)	26.22 (< 0.001)^a, b^^*^	0.28
Serial threes (/6)	4.80 (1.58)	4.31 (1.34)	5.71 (0.53)	17.95 (< 0.001)^**a, b**^	0.18
**Visuospatial skills**					
Copying of SF (/6)	5.37 (1.10)	4.45 (2.04)	5.64 (0.82)	7.07 (0.029)	0.05
Copying of SCF (/22)	20.48 (3.63)	18.16 (4.32)	21.39 (0.96)	13.65 (0.001)^b, c^^*^	0.13
CDT (/5)	4.40 (0.97)	3.45 (1.38)	4.89 (0.31)	22.72 (< 0.001)^b, c^^*^	0.24
CRT (/12)	10.0 (2.15)	8.39 (2.02)	11.05 (1.10)	27.46 (< 0.001)^b, c^^*^	0.29

Data are shown as mean (SD).

^*^Significant differences after Bonferroni correction.

^**^ANOVA was calculated for verbal fluency tasks, RPT total recall, and ECR free recall, while Kruskal Wallis test was calculated for the rest of the scores.

^**a**^Significant *post-hoc* comparison between CDR = 0.5 y HC.

^**b**^Significant *post-hoc* comparison between CDR = 1.0 y HC.

^**c**^Significant *post-hoc* comparison between CDR = 0.5 and 1.0.

^**+**^CTT 1, CDR = 0.5 sample size was 27 and CDR = 1.0 was 20; for CTT 2, CDR = 0.5 sample size was 27 and CDR = 1.0 sample size was 9.

^**++**^FDT, CDR = 1.0 sample size was 26.

RPT, Recall of pictures test; ECR, Enhanced cued recall test; SCF, Semi-complex figure; EF, Executive functions; CTT, Color trails test; FDT, Five digit test; SF, Simple figures; CDT, Clock drawing test; CRT, Clock reading test; AD-MCI, Alzheimer's disease with mild cognitive impairment group; AD-D, Alzheimer's disease with dementia; HC, Healthy control group.

Comparing AD-MCI and AD-D, scores on RPT total recall, delayed recall, and recognition, ECR total recall, recall of SCF, semantic fluency, CTT 1, copying of SCF, CDT, and CRT showed statistically significant differences, finding a lower performance in AD-D than AD-MCI.

#### 3.1.2. ROC analysis for group discrimination

For AD-MCI, AUCs of RPT, ECR, recall of SCF, and fluency tasks were higher than 0.70 and significant. All AUCs, cut-off scores, sensitivity, specificity, Youden's index, PPV, and NPV are described in Table 3. All ROC curves are represented in Supplementary material 7.

**Table 3:** ROC analysis and cutoff scores for the classification between CDR = 0.5 and HC groups.

**CNTB measure**	**AUC (CI)**	**Cutoff**	**Sensitivity (%)**	**Specificity (%)**	**Youden's index**	**PPV (%)**	**NPV (%)**
**Memory**							
RPT 1st recall	0.737 (0.602–0.863)	< 3	78.57	56.67	0.352	62.86	73.91
RPT total recall	0.746 (0.611–0.868)	< 14	85.71	53.33	0.390	63.16	80.00
RPT delayed recall	0.881 (0.695–0.918)	< 4	82.14	66.67	0.488	69.70	80.00
ECR free recall	0.893 (0.809–0.970)	< 5	75.00	83.33	0.893	80.77	78.12
ECR total recall	0.891 (0.805–0.969)	< 12	100	63.33	0.633	71.79	100
Recall of SCF	0.820 (0.709–0.931)	< 12	85.71	72.41	0.581	75.00	84.00
**Language**							
Verbal fluency “animals”	0.739 (0.607–0.866)	< 13	78.57	66.67	0.452	68.75	76.92
Verbal fluency “supermarket”	0.782 (0.659–0.899)	< 16	75.00	73.30	0.483	72.41	75.86

RPT, Recall of pictures test; ECR, Enhanced cued recall test; SCF, Semi-complex figure; AUC (CI), Area under the curve (confidence interval); PPV, Positive predictive value; NPV, Negative predictive value.

In the case of AD-D, all memory and language scores showed significant AUCs higher than 0.70. In the same line, CTT 1, FDT 3, copying of SCF, CDT, and CRT had AUCs >0.70 (Table 4).

**Table 4:** ROC analysis and cutoff scores for the classification between CDR = 1.0 and HC groups.

**CNTB measure**	**AUC (CI)**	**Cutoff score**	**Sensitivity (%)**	**Specificity (%)**	**Youden's index**	**PPV (%)**	**NPV (%)**
**Memory**							
RPT 1st recall	0.881 (0.767–0.962)	< 3	78.57	82.76	0.613	81.48	80.00
RPT total recall	0.938 (0.861–0.993)	< 13	92.86	82.76	0.756	83.87	92.31
RPT delayed recall	0.954 (0.883–1.0)	< 1	100	75.9	0.759	80.00	100
RPT recognition	0.894 (0.785–0.980)	< 9	82.14	86.21	0.683	85.19	83.33
ECR free recall	0.955 (0.896–1.0)	< 3	100	72.41	0.724	77.78	100
ECR total recall	0.975 (0.920–1.0)	< 12	100	89.66	0.897	90.32	100
Recall of SCF	0.991 (0.972–1.0)	< 6	100	96.00	0.960	96.55	100
**Language**							
Verbal fluency “animals”	0.888 (0.779–0.972)	< 12	85.71	79.31	0.650	80.00	85.19
Verbal fluency “supermarket”	0.838 (0.713–0.937)	< 16	75.00	80.00	0.550	77.78	77.42
**Attention and EF**							
CTT 1	0.786 (0.625–0.890)	>134	53.85	96.43	0.503	93.33	69.23
FDT 3	0.823 (0.676–0.925)	>71	61.54	100	0.615	100	73.68
Serial threes	0.799 (0.628–0.901)	< 5	96.43	55.17	0.516	67.50	94.12
**Visuospatial skills**							
Copying of SCF	0.759 (0.622–0.895)	< 20	96.43	56.00	0.524	71.05	93.33
CDT	0.804 (0.665–0.923)	< 4	100	55.17	0.552	68.29	100
CRT	0.895 (0.800–975)	< 10.5	85.71	85.71	0.714	85.71	85.71

RPT, Recall of pictures test; ECR, Enhanced cued recall test; SCF, Semi-complex figure; EF, Executive functions; CTT, Color trails test; FDT, Five digit test; CDT, Clock drawing test; CRT, Clock reading test; AUC (CI), Area under the curve (confidence interval); PPV, Positive predictive value; NPV, Negative predictive value.

ROC analysis comparing AD-MCI and AD-D are shown in Table 5, describing appropriate AUCs for RPT, ECR, recall of SCF, CTT 1, copying of SCF, CRT, and CDT.

**Table 5:** ROC analysis and cutoff scores for the classification between CDR = 0.5 and 1.0 groups.

**CNTB measure**	**AUC (CI)**	**Cutoff scores**	**Sensitivity (%)**	**Specificity (%)**	**Youden's index**	**PPV (%)**	**NPV (%)**
**Memory**							
RPT total recall	0.778 (0.623–0.886)	< 11	80.00	72.41	0.524	75.00	77.78
RPT delayed recall	0.730 (0.565–0.847)	< 1	66.67	75.86	0.425	74.07	68.75
RPT recognition	0.789 (0.643–0.912)	< 9	66.67	86.21	0.529	83.33	71.43
ECR total recall	0.770 (0.647–0.909)	< 9	66.67	79.31	0.460	76.92	69.70
Recall of SCF	0.818 (0.707–0.938)	< 7	62.07	96.00	0.581	94.74	68.57
**Attention and EF**							
CTT 1	0.800 (0.677–0.923)	>107	65.38	88.89	0.543	84.21	70.59
**Visuospatial skills**							
Copying of SCF	0.707 (0.550–0.846)	< 20	89.66	56.00	0.457	70.27	82.35
CDT	0.701 (0.528–0.828)	< 5	83.33	55.17	0.385	65.79	76.19
CRT	0.748 (0.509–0.862)	< 10.5	62.07	85.71	0.478	81.82	68.57

RPT, Recall of pictures test; ECR, Enhanced cued recall test; SCF, Semi-complex figure; EF, Executive functions; CTT, Color trails test; CDT, Clock drawing test; CRT, Clock reading test; AUC (CI), Area under the curve (confidence interval); PPV, Positive predictive value; NPV, Negative predictive value.

#### 3.1.3. Convergent validity between CNTB and NN scores

**Figure 1:**
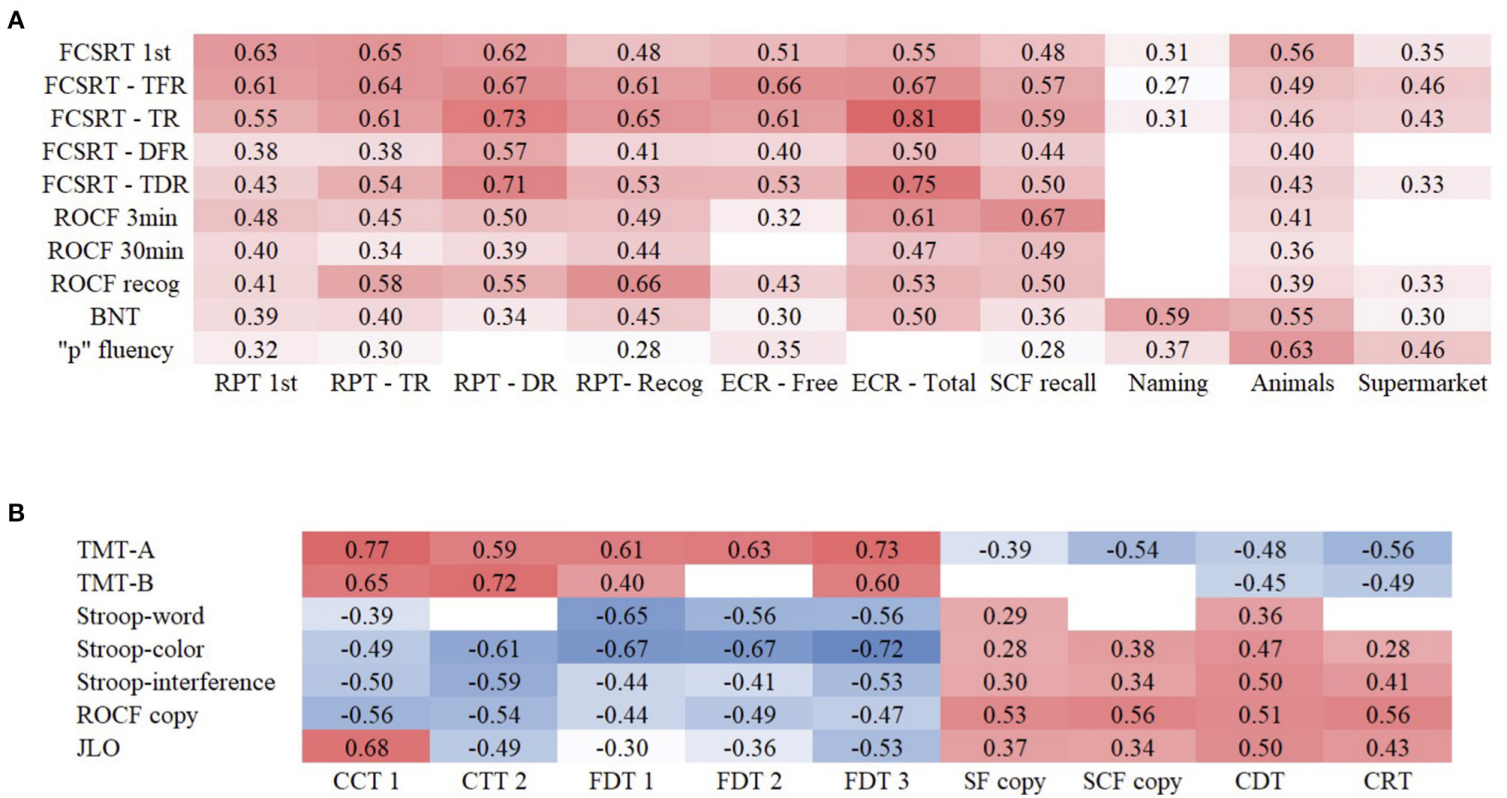
Correlations between CNTB and NN tests of memory and language **(A)** and executive functions and visuospatial functions **(B)** in AD.

Scores on RPT showed moderate correlations with the standardized neuropsychological test Free and Cued Selective Reminding Test (FCSRT). In addition, ECR scores correlated moderately and highly with FCSRT trial 1 free recall and total recall scores, respectively. Scores on copying of SCF were moderately correlated with immediate delayed recall of Rey-Osterrieth complex figure (ROCF). Similar results were found between the naming task of CNTB and Boston Naming Test (BNT) scores, and semantic fluency tasks and phonemic fluency task. The different parts of CTT and FDT were correlated with their equivalent standardized neuropsychological tests based on Trail Making test (TMT) and Stroop, respectively, showing moderate to high correlations. Visuospatial tests of CNTB displayed low to moderate correlations with Judgement of Line Orientation (JLO) and copy accuracy of ROCF. All statistically significant correlations are depicted in Figure 1.

### 3.2. Parkinson's disease with mild cognitive impairment and HC

#### 3.2.1. Intergroup comparison

We found a lower performance in PD-MCI group compared to HC in RPT, ECR, recall of SCF, semantic fluency task (supermarket), and errors of CTT 2 and FDT 3 (Table 6).

**Table 6:** Scores and differences between PD-MCI and HC groups on CNTB tests.

**CNTB measure**	**PD-MCI *n* = 30**	**HC *n* = 30**	**t^+^/U (p)**	**d^+^/r**
**Memory**				
RPT 1st recall (/10)	2.33 (1.47)	4.26 (1.37)	141.0 (< 0.001)^*^	0.31
RPT total recall (/30)	12.17 (4.43)	20.30 (4.07)	7.18 (< 0.001)^*^	1.90
RPT delayed recall (/10)	2.83 (2.42)	6.44 (2.42)	126.0 (< 0.001)^*^	0.34
RPT recognition (/10)	8.33 (1.67)	9.56 (0.80)	210.0 (< 0.001)^*^	0.18
ECR free recall (/16)	3.57 (2.11)	7.33 (2.22)	6.56 (< 0.001)^*^	1.74
ECR total recall (/16)	9.87 (3.51)	14.70 (1.17)	58.0 (< 0.001)^*^	0.52
Recall of SCF (/22)	8.31 (7.01)	17.56 (3.87)	112.0 (< 0.001)^*^	0.35
**Language**				
Naming (/10)	9.87 (0.43)	9.93 (0.27)	393.50 (0.708)	< 0.01
Verbal fluency “animals”	14.83 (5.22)	18.70 (5.81)	2.65 (0.005)	0.70
Verbal fluency “supermarket”	17.00 (5.41)	22.37 (5.79)	3.62 (< 0.001)^*^	0.96
**Attention and EF**				
CTT 1 (seconds)	90.69 (55.55)	57.63 (28.70)	532.0 (0.021)	0.09
CTT 2 (seconds)	186.21 (93.25)	136.67 (66.08)	432.5 (0.041)	0.07
CTT 2 (errors)	1.33 (1.20)	0.22 (0.58)	492.5 (< 0.001)^*^	0.22
FDT 1 (seconds)	38.17 (27.29)	27.22 (8.90)	490.5 (0.104)	0.04
FDT 2 (seconds)	38.24 (29.02)	28.70 (7.41)	498.5 (0.079)	0.05
FDT 3 (seconds)	66.48 (50.42)	45.37 (11.07)	526.5 (0.027)	0.08
FDT 3 (errors)	3.17 (2.62)	0.67 (1.04)	670.5 (< 0.001)^*^	0.39
Serial threes (/6)	5.50 (0.94)	5.70 (0.67)	367.0 (0.431)	0.01
**Visuospatial skills**				
Copying of SF (/6)	5.50 (1.07)	5.63 (0.84)	413.5 (.853)	< 0.01
Copying of SCF (/22)	20.48 (2.80)	21.48 (0.93)	350.5 (.398)	0.01
CDT (/5)	4.27 (1.08)	4.89 (0.32)	279.0 (.009)	0.11
CRT (/12)	10.10 (2.06)	11.37 (1.09)	228.5 (.005)	0.13

Data are shown was mean (SD).

^+^t-test and Cohen's d were calculated for verbal fluency tasks, RPT total recall, and ECR delayed recall, while Mann–Whitney's U and r were calculated for the rest of the scores.

^*^Significant differences after Bonferroni correction.

RPT, Recall of pictures test; ECR, Enhanced cued recall test; SCF, Semi-complex figure; EF, Executive functions; CTT, Color trails test; FDT, Five digit test; SF, Simple figures; CDT, Clock drawing test; CRT, Clock reading test; PD-MCI, Parkinson's disease with mild cognitive impairment group; HC, Healthy control group.

#### 3.2.2. ROC analysis for group discrimination

All memory scores and errors of CTT 2 and FDT 3 showed AUCs higher than 0.70 as shown in Table 7. All ROC curves are represented in Supplementary material 8.

**Table 7:** ROC analysis and cutoff scores for the classification between PD-MCI and HC.

**CNTB measure**	**AUC (CI)**	**Cutoff scores**	**Sensitivity (%)**	**Specificity (%)**	**Youden's index**	**PPV (%)**	**NPV (%)**
**Memory**							
RPT 1st recall	0.826 (0.712–0.932)	< 4	74.07	76.67	0.507	74.07	76.67
RPT total recall	0.910 (0.833–0.986)	< 17	85.19	86.67	0.719	85.19	86.67
RPT delayed recall	0.844 (0.747–0.951)	< 5	81.48	76.67	0.581	75.86	82.14
RPT recognition	0.741 (0.608–0.870)	< 10	70.37	70.00	0.404	67.86	72.41
ECR free recall	0.885 (0.804–0.970)	< 7	66.67	93.33	0.600	90.00	75.98
ECR total recall	0.759 (0.857–0.995)	< 13	92.59	83.33	0.759	83.33	92.59
Recall of SCF	0.857 (0.760–0.954)	< 12	96.30	68.97	0.653	74.29	95.24
**Attention and EF**							
CTT 2 (errores)	0.760 (0.623–0.898)	>1	62.50	85.19	0.477	78.95	71.88
FDT 3 (errores)	0.856 (0.730–0.952)	>2	72.41	85.19	0.576	84.00	74.19

RPT, Recall of pictures test; ECR, Enhanced cued recall test; SCF, Semi-complex figure; CTT, Color trails test; FDT, Five digit test; AUC (CI), Area under the curve (confidence interval); PPV, Positive predictive value; NPV, Negative predictive value.

#### 3.2.3. Convergent validity between CNTB and NN scores

**Figure 2:**
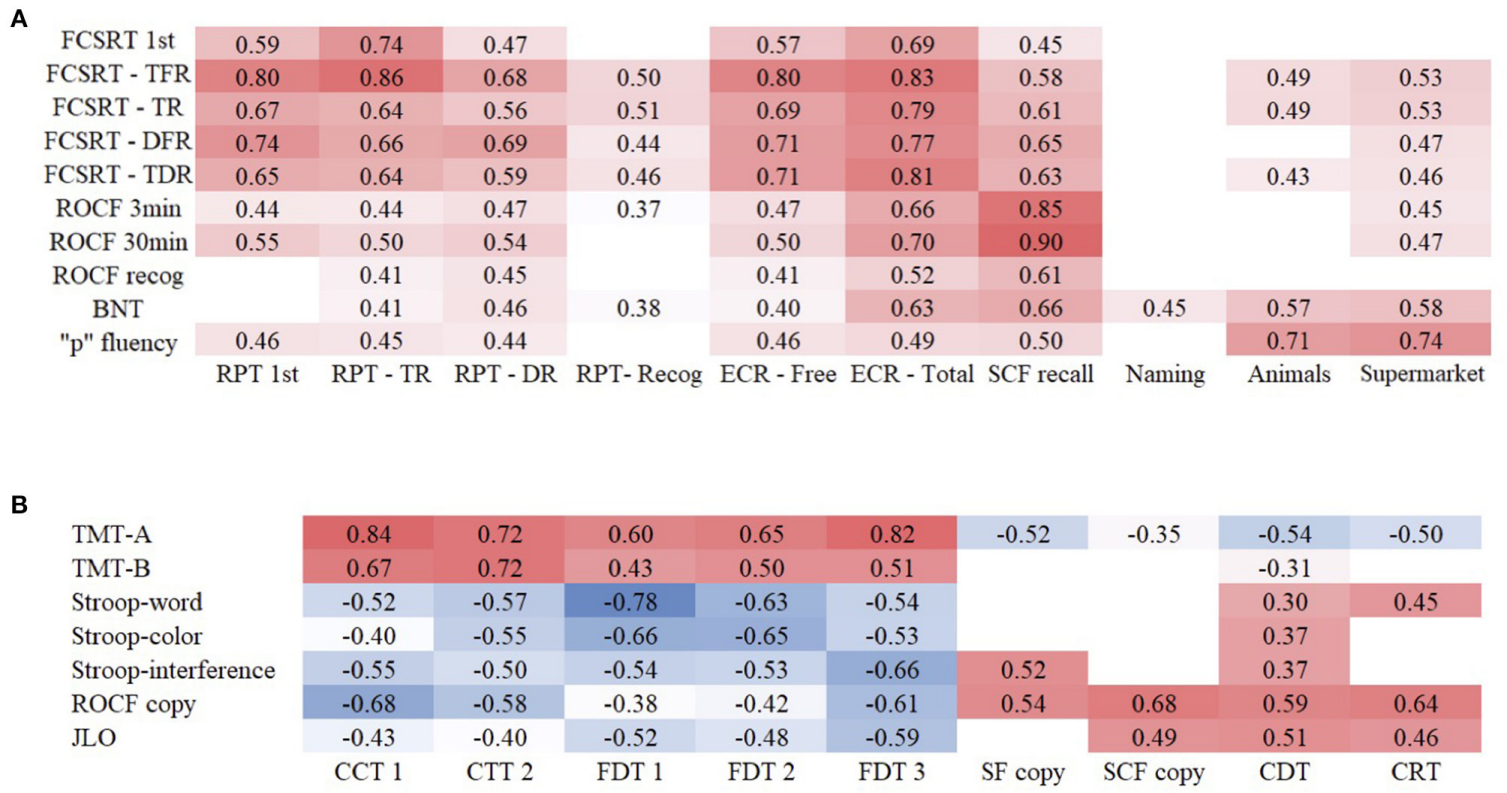
Correlations between CNTB and NN tests of memory and language **(A)** and executive functions and visuospatial functions **(B)** in PD.

The correlations between memory scores of CNTB and FCSRT scores and ROCF were moderate to high. Similarly, the semantic fluency tasks of CNTB showed high correlations with the phonemic fluency task of NN. Conversely, the naming task showed a low correlation with BNT. Scores on CCT and FDT were associated with their equivalent scores on TMT and Stroop, respectively, showing high correlations. Scores on copying of SF and SCF, CDT, and CRT correlated lowly and moderately with visuospatial standardized tests. All significant correlations are shown in Figure 2.

### 3.3. Mild cognitive impairment: AD-MCI and PD-MCI

Controlling for sex, age, and year of education, ANCOVA showed lower scores for the AD-MCI group than the PD-MCI on RPT 1st recall (*F* = 6.10, *p* < 0.001, η^2^ = 0.307), total recall (*F* = 7.35, *p* < 0.001, η^2^ = 0.348), and delayed recall (*F* = 6.95, *p* < 0.001, η^2^ = 0.336), ECR free recall (*F* = 5.12, *p* = 0.001, η^2^ = 0.279), and recall of SCF (*F* = 7.13, *p* < 0.001, η^2^ = 0.350). In contrast, PD-MCI obtained a lower performance on semantic fluency (supermarket; *F* = 7.55, *p* < 0.001, η^2^ = 0.355), CTT 1—time (*F* = 5.01, *p* = 0.002, η^2^ = 0.318), CTT 2—time (*F* = 9.73, *p* < 0.001, η^2^ = 0.475), and CTT 2—errors (*F* = 4.31, *p* = 0.005, η^2^ = 0.286) scores compared with AD-MCI.

## 4. Discussion

The CNTB has been suggested as a cross-cultural alternative to traditional neuropsychological batteries. To our knowledge, this is the first study of the CNTB in MCI due to AD and PD. In line with previous studies (Nielsen et al., 2019b; Araujo et al., 2020), the CNTB did not require any adaption to the Spanish context, supporting the cross-cultural properties of the battery.

In the case of AD groups, scores on memory were especially important for the correct classification, obtaining the largest effect sizes and AUCs higher than 0.70. In particular, RPT, ECR, recall of SCF, and semantic fluency tasks discriminated between AD-MCI and HC. In addition, the same memory and verbal fluency tests, as well as CTT 1, the most difficult part of FDT, serial threes, and some visuospatial tests were useful for distinguishing between AD-D and HC. Memory tests showed the highest effect sizes, followed by executive functions scores, according to the expected cognitive profile of AD (Leyhe et al., 2009; El Haj et al., 2015) and other cross-cultural neuropsychological tests validated in AD (Goudsmit et al., 2017; Jørgensen et al., 2019; Nielsen et al., 2019a; Delgado-Álvarez et al., 2022c).

Furthermore, these results are congruent with previous validation studies of CNTB. The first validation study found differences between the dementia group and HC on all CNTB scores, with the largest effect sizes in memory and semantic fluency tasks (Nielsen et al., 2019b). In our results, we found remarkable memory and semantic fluency deficits in AD-D, but also in AD-MCI, according to the expected cognitive profile and cognitive course of AD (Dubois et al., 2016). In addition, we found similar AUCs and cut-off scores compared to the first validation study in the AD-D group. However, AUCs of AD-MCI were lower, although our findings support the validity of the tests also in these early stages of the disease in which cognition shows lower degrees of impairment.

In a second validation study of CNTB conducted in Brazil and exclusively in a sample of AD participants at mild to moderate dementia, the authors also found high AUCs in memory tests, fluency tasks, executive functions, and visuospatial functions tests (Araujo et al., 2020), which were more similar to our AD-D sample results than AD-MCI. In our case, we did not find differences on copying of simple figures, CTT 2, or FDT 1-2 scores between AD-D and HC. However, the AD sample of Araujo et al. (2020) included more advanced stages of the disease, corresponding to CDR scores of 1.0 and 2.0. The sequential impairment of different subtests within the battery suggests the utility of CNTB as a follow-up measure in AD that may detect the most prominent deficits at each stage of the disease.

For the first time in the literature, we reported evidence of convergent validity of the CNTB as an additional source of validity in combination with construct validity, previously reported (Nielsen et al., 2018). We found moderate—high correlations between CNTB scores and scores of standardized neuropsychological tests evaluating the same cognitive domains. The only exception was the relatively low correlation (*r* = 0.43) between CRT and JLO, associated with a ceiling effect on CRT scores. While the language domain was not supported by factor analysis performed in a previous study (Nielsen et al., 2018), the naming task of the CNTB was moderately associated with scores on Boston Naming test, suggesting the assessment of the same construct. In accordance with the factor analysis (Nielsen et al., 2018), the fluency tasks (“animals” and “supermarket”) showed higher correlations with phonemic fluency scores than Boston Naming tests scores, due to the similarity between all verbal fluency tasks and also the relationship between verbal fluency and some aspects of language in combination with working memory, processing speed, and inhibition, more related to executive functions and frontal regions, as suggested in previous studies (Unsworth et al., 2011; Delgado-Álvarez et al., 2022a).

Comparing AD-D with AD-MCI, we found AUCs higher than 0.70, mainly in memory and visuospatial tests. Memory differences may reflect the different compromise of memory impairment at different stages of the disease. Similarly, visuospatial function impairment has been reported in mild dementia, but not in prodromal stage (Delgado-Álvarez et al., 2022b).

In the case of the PD-MCI group, no previous validation studies of the CNTB were conducted to compare our compare. The PD-MCI group showed a lower performance on memory and CTT and FDT errors measures than healthy controls with large effect sizes. Episodic memory deficits are frequently reported in PD, especially in recollection processes (Das et al., 2019). In this regard, the CNTB includes several episodic memory tests that showed a remarkable sensitivity to detect memory impairment in dementia of any etiology (Nielsen et al., 2019b). We found significant differences between PD-MCI and HC in some error measures, such as CTT 2 and FDT 3, which are the most demanding parts of the executive function tests, recruiting more executive function processes. This suggests the presence of attentional—executive functions impairment and not motor limitations. In fact, we did not find differences in the time measures of both tasks. In addition, we observed higher AUCs in these error scores than some memory scores. The presence of executive functioning deficits in PD-MCI in general and inhibition processes in particular has been reported in the literature (Dirnberger and Jahanshahi, 2013).

Like the AD results, we found moderate to high correlations between CNTB scores and standardized neuropsychological tests, and a low correlation between CRT and JOL, supporting the convergent validity of this battery, not only in AD, but also in PD-MCI.

Comparing the AD-MCI with the PD-MCI group, an ANCOVA model showed a lower performance of the AD-MCI than PD-MCI group on memory scores of the RPT, ECR, and delayed recall of SCF. In contrast, the PD-MCI group showed lower performance on executive functioning scores of semantic fluency, CTT (time), and CTT 2 (errors). This suggests that CNTB has the potential to capture the characteristic cognitive profile of each disorder, which paves the way for further investigation of the battery in other cognitive disorders.

Our study has some limitations. First, our study had a cross-sectional design, and conclusions about the utility of the battery for follow-up should be confirmed in longitudinal studies. In addition, although the diagnosis of AD was always supported by biomarkers, CSF biomarkers confirming amyloid and tau deposition were not available for all patients. Second, we did not include cognitively intact participants with PD for comparisons with PD-MCI. Third, no correlations between CNTB scores and clinical parameters (i.e., CSF, UPDRS, or HY) were reported. Forth, family history of dementia in the recruited patients and controls was not considered as an inclusion/ exclusion criterion. Finally, the study was powered to detect differences between groups considering large effect sizes.

In conclusion, the CNTB showed suitable diagnostic properties in AD, including prodromal stages, and in PD-MCI in a sample of Spaniards. This supports the utility of the CNTB in different clinical conditions. Any cultural or language adaptation was not required in our context. In addition, we obtained similar cut-off scores to previous studies performed in other populations, supporting the cross-cultural validity of the battery. We provided evidence of convergent validity with standardized neuropsychological tests. Overall, our findings suggest that CNTB is a valid tool to detect cognitive impairment associated with AD and PD at early stages and, intriguingly, it is able to depict the different cognitive profiles in accordance with the characteristic cognitive deficits of each disorder. Further studies in more clinical conditions and larger and more diverse populations are needed to confirm the good psychometric properties of the battery.

## Data availability statement

The raw data supporting the conclusions of this article will be made available by the authors, without undue reservation.

## Ethics statement

The studies involving human participants were reviewed and approved by CEIC Hospital Clinico San Carlos. The patients/participants provided their written informed consent to participate in this study.

## Author contributions

AD-Á: conceptualization, visualization, data curation, formal analysis, investigation, methodology, writing—original draft, and writing—review and editing. TN, JL-C, RG-R, and MG-M: investigation and writing—review and editing. CD-A, MV-S, and MD-C: data curation, investigation, and writing—review and editing. JM-G: conceptualization, visualization, funding acquisition, investigation, supervision, and writing—review and editing. JAM-G: conceptualization, visualization, data curation, formal analysis, funding acquisition, investigation, methodology, supervision, writing—original draft, and writing—review and editing. All authors contributed to the article and approved the submitted version.
