# Supplementary material for: Validation of the European Cross-Cultural Neuropsychological Test Battery (CNTB) for the assessment of mild cognitive impairment due to Alzheimer's disease and Parkinson's disease
Source: Front Aging Neurosci. 2023 May 5;15:1134111. doi: 10.3389/fnagi.2023.1134111 (PMC10196233; doi:10.3389/fnagi.2023.1134111)
Supplement: Supplementary file 1 [file Data_Sheet_1.pdf]

Supplementary Material

Supplementary Material 1. Comparisons between clinical groups and their control groups.

Supplementary Material 2. Brief description of CNTB tests.

Supplementary Material 3. Predictive validity estimates at different base rates of CDR = 0.5.

Supplementary Material 4. Predictive validity estimates at different base rates of CDR = 1.0.

Supplementary Material 5. Predictive validity estimates at different base rates of CDR = 1.0 and CDR = 0.5.

Supplementary Material 6. Predictive validity estimates at different base rates of PD-MCI.

Supplementary Material 7. ROC curves for all CNTB tests are divided into memory, executive functions, and language & visuospatial functions in AD-MCI (A) and AD-D (B).

Supplementary Material 8. ROC curves for all CNTB tests are divided into memory, executive functions, and language & visuospatial functions in PD-MCI

Supplementary Material 1. Comparisons between clinical groups and their control groups.

Group	Sex	Age	Years of education
AD vs. HC	X^2= 1.659, p = .436	F = 0.567, p = .569	H = 1.17, p = .557
PD-MCI vs. HC	X^2 = 3.590, p = .103	t = -1.068, p = .290	U = 568.5, p = .076
