# Supplementary material for: Validation of the European Cross-Cultural Neuropsychological Test Battery (CNTB) for the assessment of mild cognitive impairment due to Alzheimer's disease and Parkinson's disease
Source: Front Aging Neurosci. 2023 May 5;15:1134111. doi: 10.3389/fnagi.2023.1134111 (PMC10196233; doi:10.3389/fnagi.2023.1134111)
Supplement: Supplementary file 2 [file Data_Sheet_2.PDF]

Supplementary Material 2. Brief description of CNTB tests.

Test	Description	Scores
**Memory**		
Recall of Pictures Test (RPT)	Episodic memory task with 10 pictures to be remembered on three trials, followed by a delayed free recall and a recognition task after 10 minutes of distraction.	1st recall: 10<br>Total recall: 30<br>Delayed free recall: 10<br>Recognition task: 10
Enhanced Cued Recall test	Similar to the first trial of the Free and Cued Selective Reminding test. After the presentation of 10 pictures, participants are asked to freely recall all of them, followed by a cued recall for all items not freely recalled.	Free recall: 16<br>Cued recall: 16<br>Total recall: 16
Recall of semi-complex figure	Free recall of the semi-complex figure that was copied 3 minutes ago.	Total score: 22
**Language**		
Picture naming	Naming task during the presentation of RPT stimuli	Total score: 10
Semantic fluency tasks	Two fluency tasks of 1 minute for animals and supermarket	Correct words
**Executive functions**		
Color Trails Test (CTT)	Similar to the Trail Making test. Participants are asked to connect numbered circles in ascending order (CTT1) and to switch pink and yellow (e.g., 1 pink, 2 yellow, 3 pink, etc) (CTT2).	Time in seconds for each part

Five Digit Test (FDT)	Similar to the Stroop test. During FDT 1, participants are required to name a series of 50 digits; during FDT 2, they have to count a series of 50 asterisks; and during FDT 3 they have to count (and not name) a series of 50 digits.	Time in seconds for each part
Serial threes	Participants are asked to count down from 20 by threes.	Total score: 6
**Visuospatial functions**		
Copying of simple figures	Copy of a Greek cross and a four-pointed star	Total score: 6
Copying of semi-complex figure	Copy of semi-complex figure.	Total score: 22
Clock Drawing Test (CDT)	Participants are asked to insert numbers and indicate 11:10 in a pre-drawn circle	Total score: 5
Clock Reading Test (CRT)	Participants are required to read the time on 12 different clocks without numbers	Total score: 12
