# Supplementary material for: Validation of the European Cross-Cultural Neuropsychological Test Battery (CNTB) for the assessment of mild cognitive impairment due to Alzheimer's disease and Parkinson's disease
Source: Front Aging Neurosci. 2023 May 5;15:1134111. doi: 10.3389/fnagi.2023.1134111 (PMC10196233; doi:10.3389/fnagi.2023.1134111)
Supplement: Supplementary file 3 [file Data_Sheet_3.PDF]

Supplementary Material 3. Predictive validity estimates at different base rates of CDR = 0.5.

CNTB measure	Cutoff	Primary care: base rate 25%		Memory clinics: base rate 50%	
		PPV (95% CI)	NPV (95% CI)	PPV (95% CI)	NPV (95% CI)
**Memory**					
RPT 1st recall	< 3	37.67% (27.76 – 48.73)	88.81% (78.51 – 94.51)	64.45% (53.56 – 74.03)	72.56% (54.91 – 85.17)
RPT total recall	< 14	37.97% (28.86 – 48.02)	91.80% (80.98 – 96.72)	64.75% (54.90 – 73.48)	78.87% (58.67 – 90.76)
RPT delayed recall	< 4	45.10% (32.49 – 58.37)	91.80% (82.95 – 96.27)	71.13% (59.08 – 80.79)	78.87% (61.86 – 89.58)
ECR free recall	< 5	60.00% (39.59 – 77.45)	90.91% (83.77 – 95.09)	81.82% (66.28 – 91.15)	76.92% (63.25 – 86.59)
ECR total recall	< 12	47.62% (36.23 – 59.27)	100%	73.17% (63.02 – 81.36)	100%
Recall of SCF	< 12	50.88% (36.04 – 65.56)	93.83% (85.66 – 97.48)	75.65% (62.83 – 85.10)	83.52% (66.56 – 92.81)
**Language**					
Verbal fluency "animals"	< 13	44.00% (31.37 – 57.46)	90.32% (81.47 – 95.20)	70.21% (57.83 – 80.21)	75.68% (59.43 – 86.85)
Verbal fluency "supermarket"	< 16	48.39% (33.29 – 63.79)	89.80% (81.73 – 94.54)	73.77% (59.95 – 84.09)	74.58% (59.85 – 85.23)

RPT = Recall of Pictures Test, ECR = Enhanced Cued Recall test, SCF = semi-complex figure, AUC (CI) = area under the curve (confidence interval), PPV = positive predictive value, NPV = negative predictive value.

Supplementary Material 4. Predictive validity estimates at different base rates of CDR = 1.0.

CNTB measure	Cutoff	Primary care: base rate 25%		Memory clinics: base rate 50%	
		PPV (95% CI)	NPV (95% CI)	PPV (95% CI)	NPV (95% CI)
**Memory**					
RPT 1st recall	< 3	60.30% (40.07 – 77.53)	92.05% (84.83 – 96.00)	82.01% (66.73 – 91.19)	79.43% (65.08 – 88.89)
RPT total recall	< 13	64.22% (44.55 – 80.05)	97.20% (90.05 – 99.26)	84.34% (70.68 – 92.33)	92.05% (75.10 – 97.80)
RPT delayed recall	< 1	58.00% (42.01 – 72.47)	100%	80.56% (68.48 – 88.76)	100%
RPT recognition	< 9	66.50% (44.02 – 83.37)	93.54% (86.59 – 97.01)	85.62% (70.23 – 93.76)	82.84% (68.28 – 91.54)
ECR free recall	< 3	54.72% (40.12 – 68.54)	100%	78.38% (66.78 – 86.73)	100%
ECR total recall	< 12	76.32% (52.46 – 90.39)	100%	90.62% (76.80 – 96.58)	100%
Recall of SCF	< 6	89.29% (54.98 – 98.27)	100%	96.15% (78.56 – 99.42)	100%
**Language**					
Verbal fluency “animals”	< 12	58.00% (39.99 – 74.10)	94.34% (86.84 – 97.68)	80.56% (66.66 – 89.57)	84.74% (68.74 – 93.34)
Verbal fluency “supermarket”	< 16	55.56% (37.20 – 72.51)	90.57% (83.14 – 94.92)	78.95% (63.99 – 88.78)	76.19% (62.18 – 86.17)
**Attention & EF**					
CTT 1	>134	71.53% (38.68 – 90.92)	85.79% (79.74 – 90.25)	88.29% (65.43 – 96.78)	66.80% (56.74 – 75.52)
FDT 3	> 71	100%	88.64% (82.75 – 92.69)	100%	72.22% (61.52 – 80.87)
Serial threes	< 5	41.76% (32.24 – 51.93)	97.89% (86.80 – 99.69)	68.27% (58.81 – 76.42)	93.92% (62.63 – 86.16)
**Visuospatial skills**					
Copying of SCF	< 20	42.21% (31.82 – 53.34)	97.92% (86.93 – 99.70)	68.67% (58.34 – 77.43)	94.00% (68.92 – 99.11)

CDT	< 4	42.65% (33.18 – 52.69)	100%	69.05% (59.83 – 76.96)	100%
CRT	< 10.5	66.67% (44.36 – 83.38)	94.74% (87.77 – 97.83)	85.71% (70.51 – 93.77)	85.71% (73.78 – 93.62)

RPT = Recall of Pictures Test, ECR = Enhanced Cued Recall test, SCF = semi-complex figure, EF = executive functions, CTT = Color Trails Test, FDT = Five Digit Test, CDT = Clock Drawing Test, CRT = Clock Reading Test, AUC (CI) = area under the curve (confidence interval), PPV = positive predictive value, NPV = negative predictive value.

Supplementary Material 5. Predictive validity estimates at different base rates of CDR = 1.0 and CDR = 0.5.

CNTB measure	Cutoff	Primary care: base rate 25%		Memory clinics: base rate 50%	
		PPV (95% CI)	NPV (95% CI)	PPV (95% CI)	NPV (95% CI)
**Memory**					
RPT total recall	< 11	49.15% (34.30 – 64.16)	91.57% (83.69 – 95.83)	74.36% (61.03 – 84.30)	78.36% (63.10 – 88.46)
RPT delayed recall	< 1	47.93% (31.52 – 64.80)	87.22% (79.82 – 92.18)	73.42% (58.00 – 84.67)	69.47% (56.86 – 79.71)
RPT recognition	< 9	61.70% (38.52 – 80.56)	88.58% (82.09 – 92.93)	82.86% (65.27 – 92.55)	72.12% (60.43 – 81.41)
ECR total recall	< 9	51.79% (33.52 – 69.59)	87.71% (80.63 - 92.45)	76.32% (60.20 – 87.28)	70.41% (58.12 – 80.31)
Recall of SCF	< 7	73.19% (41.31 – 91.37)	88.89% (82.71 – 93.05)	89.12% (67.86 – 96.95)	72.74% (61.46 – 81.70)
**Attention & EF**					
CTT 1	>107	66.23% (39.43 – 85.53)	88.51% (81.71 – 93.00)	85.47% (66.14 – 94.66)	71.97% (59.83 – 81.58)
**Visuospatial skills**					
Copying of SCF	< 20	40.45% (30.03 – 51.81)	94.20% (84.04 – 98.04)	67.08% (56.28 – 76.33)	84.41% (63.70 – 94.35)
CDT	< 5	38.26% (28.64 – 48.89)	90.85% (80.70 – 95.93)	65.02% (54.63 – 74.16)	76.80% (58.23 – 88.71)
CRT	< 10.5	59.15% (35.88 – 78.94)	87.15% (80.60 – 91.71)	81.29% (62.67 – 91.83)	69.32% (58.07 – 78.66)

RPT = Recall of Pictures Test, ECR = Enhanced Cued Recall test, SCF = semi-complex figure, EF = executive functions, CTT = Color Trails Test, CDT = Clock Drawing Test, CRT = Clock Reading Test, AUC (CI) = area under the curve (confidence interval), PPV = positive predictive value, NPV = negative predictive value.

Supplementary Material 6. Predictive validity estimates at different base rates of PD-MCI.

**CNTB measure**	**Cutoff**	**Primary care: base rate 25%**		**Memory clinics: base rate 50%**	
		PPV (95% CI)	NPV (95% CI)	PPV (95% CI)	NPV (95% CI)
**Memory**					
RPT 1st recall	< 4	51.41% (34.76 – 67.75)	89.87% (81.99 – 94.53)	76.05% (61.52 – 86.31)	74.73% (60.27 – 85.22)
RPT total recall	< 17	68.05% (45.76 – 84.31)	94.61% (87.54 – 97.77)	86.47% (71.68 – 94.16)	85.40% (70.08 – 93.59)
RPT delayed recall	< 5	53.79% (37.26 – 69.53)	92.55% (84.60 – 96.56)	77.74% (64.05 – 87.25)	80.54% (64.68 – 90.35)
RPT recognition	< 10	43.88% (30.05 – 58.73)	87.64% (79.11 – 92.99)	70.11% (56.31 – 81.02)	70.26% (55.80 – 81.55)
ECR free recall	< 7	76.92% (45.98 – 92.89	89.36% (83.01 – 93.52)	90.91% (71.86 – 97.51)	73.68% (61.96 – 82.80)
ECR total recall	< 13	64.94% (45.24 – 80.59)	97.12% (89.81 – 99.23)	84.75% (71.25 – 92.57)	91.84% (74.60 – 97.73)
Recall of SCF	< 12	50.84% (37.43 – 64.14)	98.24% (88.93 – 99.74)	75.63% (64.22 – 84.29)	94.90% (72.82 – 99.23)
**Attention & EF**					
CTT 2 (errores)	> 1	58.44% (35.09 – 78.53)	87.20% (79.89 – 92.12)	80.84% (61.86 – 91.65)	69.43% (56.97 – 79.58)
FDT 3 (errores)	> 2	62.54% (39.76 – 80.85)	90.83% (84.23 – 94.83)	83.36% (66.44 – 92.68)	76.75% (64.04 – 85.95)

RPT = Recall of Pictures test, ECR = Enhanced Cued Recall test, SCF = semi-complex figure, CTT = Color Trails Test, FDT = Five Digit Test, AUC (CI) = area under the curve (confidence interval), PPV = positive predictive value, NPV = negative predictive value.
