# Supplementary figures and images for: Validation of the European Cross-Cultural Neuropsychological Test Battery (CNTB) for the assessment of mild cognitive impairment due to Alzheimer's disease and Parkinson's disease
Source: Front Aging Neurosci. 2023 May 5;15:1134111. doi: 10.3389/fnagi.2023.1134111 (PMC10196233; doi:10.3389/fnagi.2023.1134111)

**A**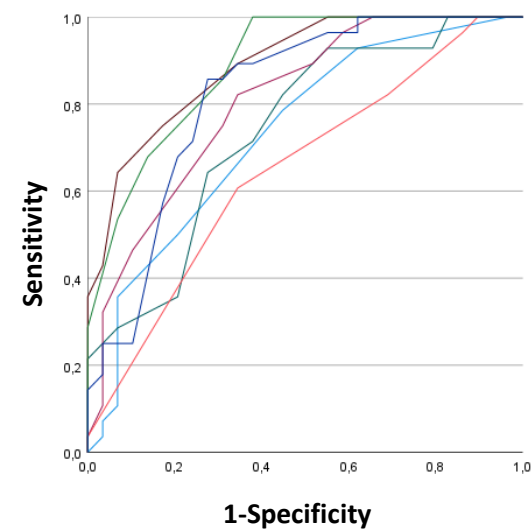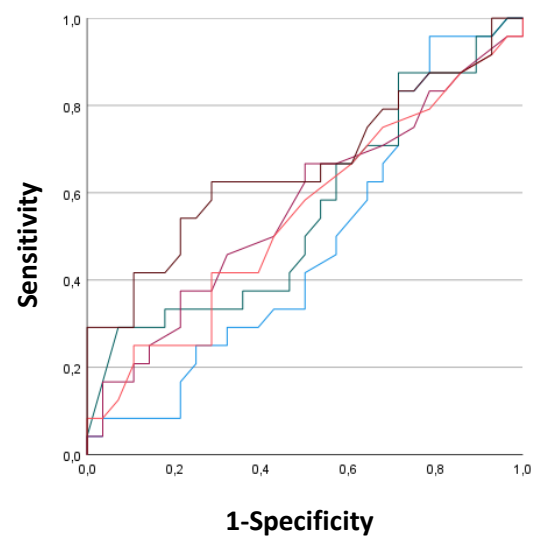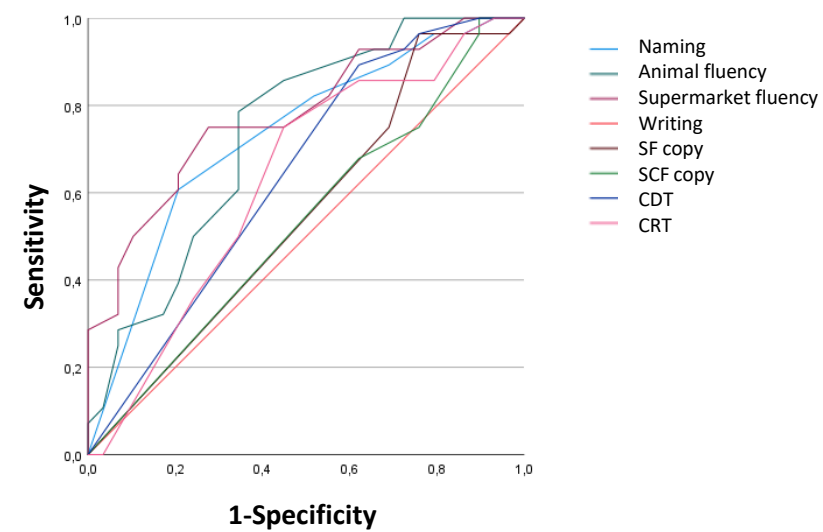**B**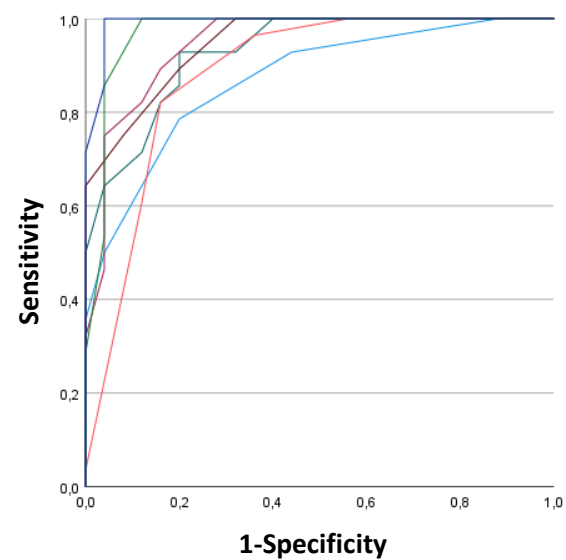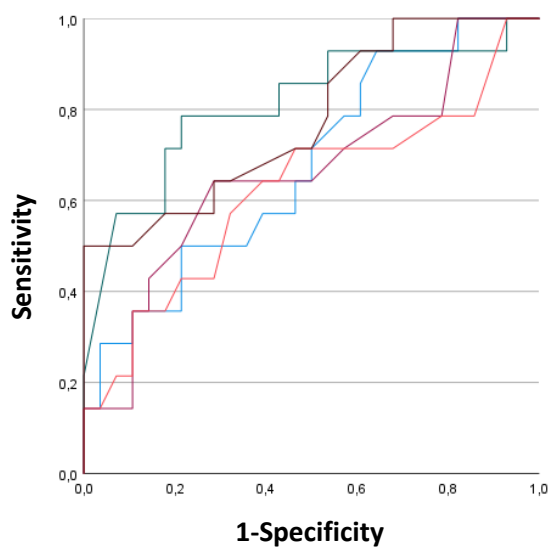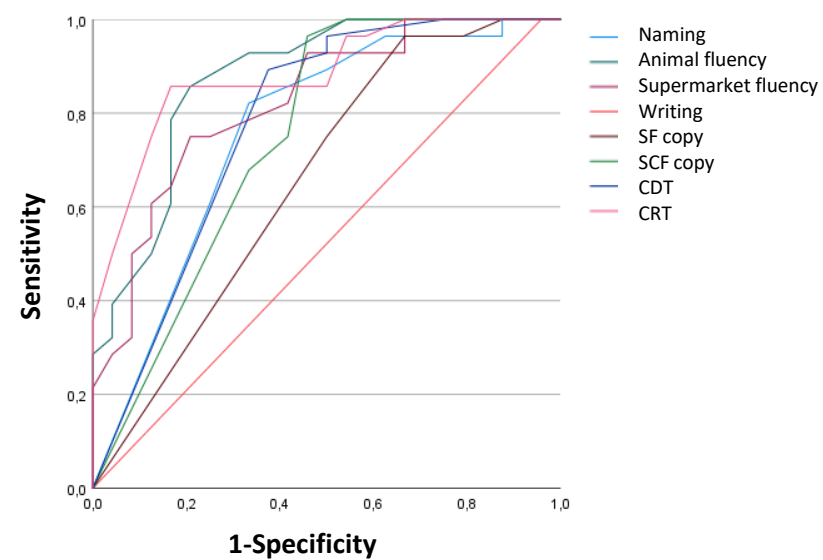

Supplement: Supplementary file 4 [file Data_Sheet_4.PDF]

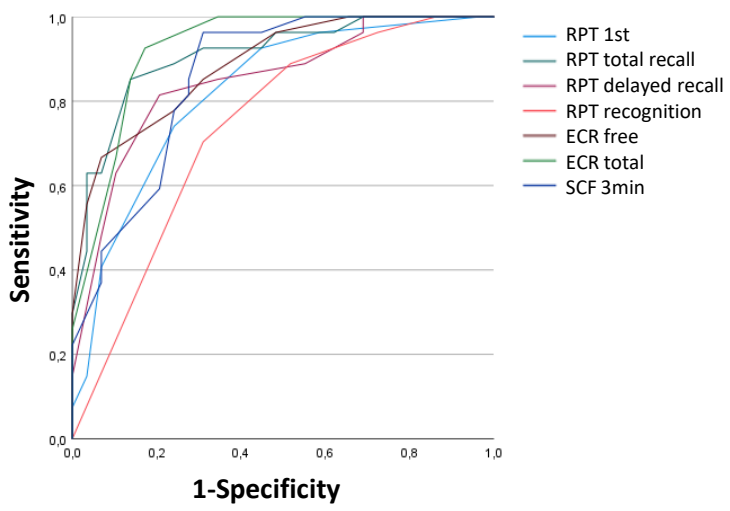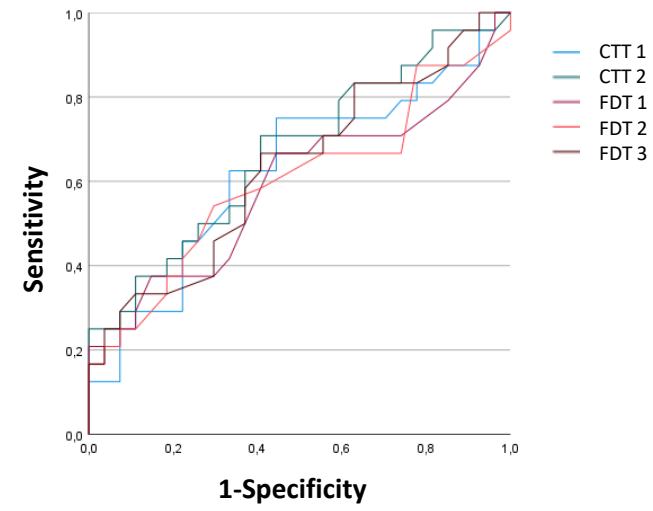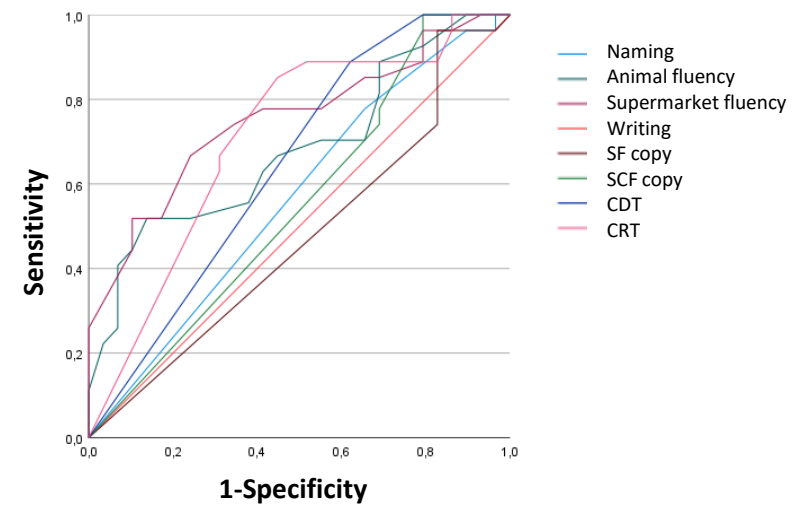

Supplement: Supplementary file 5 [file Data_Sheet_5.PDF]
